# Mechanism of Impact of Big Data Resources on Medical Collaborative Networks From the Perspective of Transaction Efficiency of Medical Services: Survey Study

**DOI:** 10.2196/32776

**Published:** 2022-04-21

**Authors:** Junyi Yuan, Sufen Wang, Changqing Pan

**Affiliations:** 1 Information Center Shanghai Chest Hospital Shanghai Jiao Tong University Shanghai China; 2 Department of Management Science and Engineering Glorious Sun School of Business & Management Donghua University Shanghai China; 3 Hospital’s Office Shanghai Chest Hospital Shanghai Jiao Tong University Shanghai China

**Keywords:** medical collaborative networks, big data resources, transaction efficiency

## Abstract

**Background:**

The application of big data resources and the development of medical collaborative networks (MCNs) boost each other. However, MCNs are often assumed to be exogenous. How big data resources affect the emergence, development, and evolution of endogenous MCNs has not been well explained.

**Objective:**

This study aimed to explore and understand the influence of the mechanism of a wide range of shared and private big data resources on the transaction efficiency of medical services to reveal the impact of big data resources on the emergence and development of endogenous MCNs.

**Methods:**

This study was conducted by administering a survey questionnaire to information technology staff and medical staff from 132 medical institutions in China. Data from information technology staff and medical staff were integrated. Structural equation modeling was used to test the direct impact of big data resources on transaction efficiency of medical services. For those big data resources that had no direct impact, we analyzed their indirect impact.

**Results:**

Sharing of diagnosis and treatment data (*β*=.222; *P*=.03) and sharing of medical research data (*β*=.289; *P*=.04) at the network level (as big data itself) positively directly affected the transaction efficiency of medical services. Network protection of the external link systems (*β*=.271; *P*=.008) at the level of medical institutions (as big data technology) positively directly affected the transaction efficiency of medical services. Encryption security of web-based data (as big data technology) at the level of medical institutions, medical service capacity available for external use, real-time data of diagnosis and treatment services (as big data itself) at the level of medical institutions, and policies and regulations at the network level indirectly affected the transaction efficiency through network protection of the external link systems at the level of medical institutions.

**Conclusions:**

This study found that big data technology, big data itself, and policy at the network and organizational levels interact with, and influence, each other to form the transaction efficiency of medical services. On the basis of the theory of neoclassical economics, the study highlighted the implications of big data resources for the emergence and development of endogenous MCNs.

## Introduction

### Background

There has been a long-term coexistence of imbalanced allocation and low use efficiency of medical resources in China. Most health care reforms have tried to encourage a variety of medical collaborative practices as a means to improve the quality and efficiency of health care delivery. For example, the New Rural Cooperative Medical Scheme was launched to protect rural households from catastrophic medical expenditure [1] and various medical consortia were mainly used to improve the system of tiered medical services to balance inadequate medical resources [2]. These studies often assumed that medical collaborative networks (MCNs) are exogenous and had already been formed. However, many medical collaborative practices have not achieved the desired results. Su et al [3] showed that there was no statistically significant difference between the distribution of inpatients in county and township hospitals before and after the implementation of the New Rural Cooperative Medical Scheme in China. The practice of collaborative health care will produce various forms of MCNs. The MCNs’ structures are always complex [4]. It was corroborated that the MCNs’ structures and collaborative practices influence each other [5], the mutual recursive influence becoming meaningful through a complex net of organizational and institutional features, as well as patients’ nosological profiles [6]. MCNs are often assumed to be exogenous; however, they are endogenous. It is very important to pay attention to how endogenous MCNs emerge and develop.

At the same time, the development of the internet and big data technology has promoted the transformation of medical service patterns and management modes [7,8], leading to the emergence of various MCNs, such as collaboration between hospitals of different levels [2,9]. Furthermore, many internet companies (such as Hao Daifu, Chunyu Doctor, and Weiyi) have been pouring into the medical service industry to lead more diverse forms of medical collaborative practices [10]. Big data resources in health care have advanced the development of MCNs, which in turn further promotes the application of big data in the health care field [11]. It is generally believed that big data resources affect the emergence and development of MCNs; yet, there is a lack of understanding of the mechanism of the impact of big data resources on the emergence and development of MCNs.

As the organizational network has increasingly become an important form of business operation, the commercial value of information technology (IT) to the organizational network has gradually become an issue of concern. Han et al [12] analyzed the value of the relationship, based on the enterprise resource planning system, between suppliers of the enterprise resource planning system and their partners through case studies. Ceccagnoli et al [13] explored the cocreation of value in a platform ecosystem based on the resource-based view of the firm. These studies have emphasized the organizational privatization of traditional IT resources [14,15] without considering the particularity of big data resources or the coexistence of shared and private resources in the organizational network [16]. The value realization of big data should be analyzed from the work practice, organizational, and supraorganizational levels [17] and be integrated information, technology, policy, and so on [18,19].

This study aims to explore and understand the influence of the mechanism of shared and private big data resources on the emergence and development of MCNs. The coexistence of labor division and cooperation is not only the most basic phenomenon of MCNs, but also the most basic driving force of survival and development. On the basis of neoclassical economics, this paper took the transaction efficiency of medical services as a key variable to represent the emergence and development of endogenous MCNs. Next, we classified big data resources related to value cocreation of MCNs according to two dimensions: (1) public big data resources at the network level versus private big data resources at the medical institution level and (2) the three elements of big data value (data itself, technology, and various organizational elements). At the level of medical institutions in the MCN, there are external web-based big data (health care big data itself) and outward interaction security (big data technology); at the public level of the MCN, there are sharing of big data (health care big data itself) and policies and regulations related to big data (data policy). Finally, we empirically analyzed the direct and intermediary effects of all kinds of big data resources on the transaction efficiency of medical services.

### Hypotheses and Modeling

#### Transaction Efficiency of Medical Services

Medical collaboration refers to a process that occurs when a group of autonomous stakeholders with various medical resources communicate and coordinate with each other to share decision-making, goal setting, and implementation of a plan of care [2,5,6,20].

Extant empirical studies often assumed that MCNs are exogenous and found that medical collaborative practices may be affected by factors at individual, organizational, and system levels, such as mutual trust [20,21], IT infrastructure [22-24], medical policies, investment of public funds [9], and remuneration methods [4]. However, the conclusions drawn regarding the influence of these factors are inconsistent and contradictory [5]. Because of the interaction of many factors, it is necessary to analyze the nature of the impact of these factors on medical collaborative practices from the perspective of system and process [5,6].

From the perspective of system and process, various forms of medical collaborative practices have been explored. Touati et al [6] elicited three specific modalities of collaboration: quasi-inexistent, restrained, and extended. Braun and Cusick [25] explored four innovative care models that aimed to expand access to dental care: expanded coordinated care, colocated care, integrated care, and virtual dental home. Huang and Li [26] divided the medical alliance into three types (compact, semicompact, and loose) according to the closeness of the contact. The recursive interaction between structures and collaborative practices has been corroborated [5] and becomes meaningful through a complex network of organizational and institutional characteristics and the nosological profiles of patients [6]. However, to explore the influence of the mechanism of big data resources on the emergence and development of MCNs, we need to integrate factor research and structure research to determine a theoretical construct that can reflect the changes in network structure and embody various factors influencing collaborative practices.

The neoclassical economics framework proposed by Yang and Ng [27] studied organizational topological properties by introducing transaction costs. The increase in division of labor will increase the number of transactions, and each transaction will produce transaction costs. If the transaction efficiency is low, the transaction cost is greater than the specialized economy generated by the division of labor and individuals will choose to be self-sufficient. If the transaction efficiency is fully improved, the transaction cost is offset by the specialized economy and individuals will choose division of labor. Therefore, organizational topological properties are closely related to transaction efficiency: the smaller the size of the organization, the more the cooperation with the outside world [28].

MCN members are afforded both cooperation and division of labor. Touati et al [6] emphasized that transaction cost cannot be ignored in all kinds of collaboration involving various factors at individual, organizational, and clinical levels. Collaborative practice requires collaborators to share rules, beliefs, and codes of conduct [5], regarding which there are often differences in the collaborators’ cognitions. These differences will incur transaction costs, affecting the results of collaborative practice. McComb et al [20] showed that physicians and nurses in general medical units have different perceptions of role, responsibility, and mutual trust, which act as obstacles to cooperation in these units. Communication problems among collaborators often persist and seriously affect the implementation of collaborative practices. Without videoconferencing, some diagnostic pathways (visual and clinical examination) would be lost in the interaction between cardiologists and family physicians [23]. The traditional written referral usually led to incomplete information, thus affecting the quality and comprehensiveness of communication [24]. There are also some factors at the system level, such as poor public infrastructure [9], that lead to low transaction efficiency and high transaction cost.

Because of the characteristics of autonomy and limited resources, there is division of labor everywhere in MCNs. At the same time, the collaborative community is different from the simple addition of the original individuals and relies on value rationality among members to create a unique social structure oriented to the ultimate goal of common commitment, which can support members to work collaboratively [29]. The decision of whether to choose medical collaborative practice is based on the trade-off between the health care specialized economy and transaction cost. Collaborators make decisions in their own self-interest under a specific MCN, but their decisions are affected by other decision-makers in the MCN. Finally, through the interaction of all parties and the balance of interests, a specific structure will emerge. The MCN’s structure and individual decision-making are entangled to produce and reproduce. To sum up, MCNs are endogenous and the transaction efficiency of health care is the key variable for the emergence and development of MCNs.

In this paper, the transaction efficiency of medical services refers to the quality of the medical transaction service. The higher the quality of the transaction service, the smaller the transaction cost and the higher the transaction efficiency. At this time, it is more likely that MCNs will be chosen to provide medical services in a cooperative way.

#### Big Data Resources

There were 2 main concepts of big data. The first is based on the characteristics of the generated data, such as the 3V model [30], 4V model [31], and 5V model [32]. The second is focused on various technologies and methods such as big data storage and management [33], cloud computing and cloud service [34], big data security and privacy [31], real-time data-processing technology [7], and various big data analysis technologies [35]. De Mauro et al [18] proposed that the four elements (technology, method, information, and impact) that affect the value of big data should be integrated. Wamba et al [19] believed that the business value of big data is enabled through data policy, technology, organizational change, data access, industry structure, and so on. However, the classification of these value factors lack a theoretical basis.

The IT resources of a single organization were often conceptualized and classified based on the resource-based view of the firm [14,15], which emphasized the organizational privatization of resources with a clear definition of property rights. Dover [16] studied the business value of IT based on the relationship theory, expanded the limitations of the resource-based view of the firm on the assumption of ownership and control of resources, and distinguished shared resources from nonshared resources. In network organizations, IT resources (especially big data resources) are both publicly owned by the network and privately owned by a specific organization.

We applied and further extended the classification of IT resources for a single organization [14,15] to that of big data for MCNs and extended the process of realizing IT business value to the process of realizing big data business value. Big data resources for MCNs involve health care big data itself, big data technology, and data policy at both the public level of MCNs and the institution level in MCNs. At the level of medical institutions in the MCN, external web-based big data (health care big data itself) and outward interaction security (big data technology) form the conditions and basis for medical institutions to export or import medical services as decision-makers. At the public level of MCNs, the sharing of big data (health care big data itself) and policies and regulations related to big data (data policy) affect all kinds of support conditions and constraints for the operation of medical institutions in MCNs by forming or changing the public environment at the network level.

At the level of medical institutions in the MCN, external web-based big data resources (big data itself) play a balancing and optimizing role in ensuring the supply of medical service resources to other hospitals or institutions and include real-time data of diagnosis and treatment services and medical service capacity available for external use. Real-time data of diagnosis and treatment services refers to the degree to which a medical institution provides information on physician suspending the diagnosis and treatment and opening consultations for external systems (such as remote consultation platforms, government public platforms, and medical networking). Real-time data of diagnosis and treatment services are the data source of the catalog of external services provided by medical institutions [36,37]. Medical service capacity available for external use is a medical institution’s ability to determine medical service resources such as consultation services and appointment services that can be provided to other hospitals or institutions and can be obtained by comparing the real-time use status of the medical service resources with the ideal status [38,39]. Medical service capacity available for external use is a relevance index of health care big data that reflects the connectivity of health care data [40,41].

As big data technology, outward interaction security at the level of medical institutions provides security for stable and continuous connection of data distributed at different medical institutions. It includes encryption security of web-based data and network protection of external link systems. Encryption security of web-based data is the perceived ability of a medical institution to ensure data security during interaction with other hospitals or institutions [42,43]. Network protection of the external link systems is the perceived ability of a medical institution to deploy the physical security foundation for the connection between medical institutions and the outside world [24,44].

At the public level of MCNs, sharing of big data may improve medical service and research capabilities by sharing health care big data with each other [45]. This includes the sharing of diagnosis and treatment data as well as medical research data. Sharing of diagnosis and treatment data refers to the degree to which a medical institution within MCNs can obtain diagnosis and treatment data from other medical institutions through government public platforms or third-party platforms [41,46]. Sharing of research data refers to the degree to which a medical institution within MCNs can obtain research data from other medical institutions through Chinese National Knowledge Infrastructure, PubMed, and so on. Policies and regulations related to big data at the public level of MCNs refers to the degree to which policies, laws, and regulations (such as 3-level referral from the Health and Family Planning Commission, medical consortium, and regional medical treatment center) can support the construction of the regional medical service platform [19].

#### Model

On the basis of the assumption that MCNs are endogenous and that the transaction efficiency of health care is the key variable for the emergence and development of MCNs, this study aims to explore and understand the mechanism of the influence of shared and private big data resources in MCNs on transaction efficiency to reveal the impact of big data resources on the emergence and development of MCNs. The research questions are as follows:

1. What big data resources at the two levels (shared and private) directly affect transaction efficiency?
2. When there is no direct impact, what are the paths of indirect influence of these big data resources on transaction efficiency?

**Figure 1:**
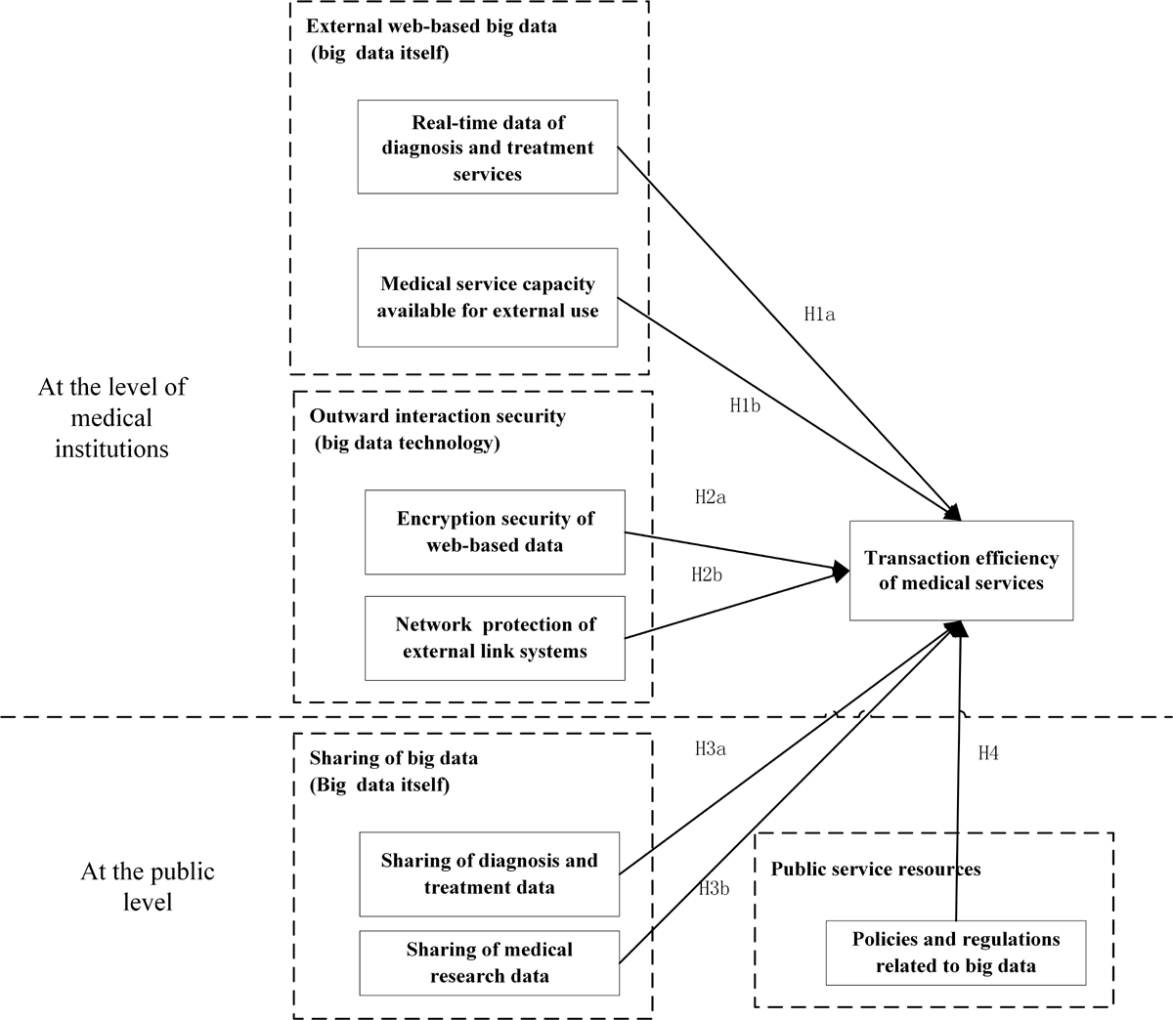
Conceptual model: the impact of big data resources of medical collaborative networks on transaction efficiency of medical services. H: hypothesis.

Figure 1 presents the model examined in this research. It shows relationships that are hypothesized to exist among big data resources at the level of medical institutions in the MCN, big data resources at the public level of the MCN, and the transaction efficiency of medical services.

## Methods

### Measurement Instruments and Questionnaire Development

#### Overview

For most constructs, measures validated in previous studies were adapted. For constructs unique to the model, multiple operational measures based on field interviews were developed. All constructs were measured using a 7-point Likert-scale ranging from 1=*strongly disagree* to 7=*strongly agree*. Details of the measures are presented in Multimedia Appendix 1.

#### Transaction Efficiency of Medical Services

In this study, collaborative medical care was mainly carried out through third-party platforms such as Baiyulan and cloud hospitals. The transaction efficiency of medical services depends on the quantity and quality of medical service resources provided by the platform. The first concerns the scope and level of experts available on the platform. The accuracy, real-time nature, and comprehensiveness of information on the experts enable the requester to know the experts in time, make correct judgments, and reduce unnecessary transaction costs caused by the provision of asymmetric information. The second concerns the performance of the communication mechanism provided by the platform for all partners. To better cooperate with collaborative diagnosis and treatment, the platform needs to support multiple medical institutions to read medical records and images on the web and in real time at the same time to ensure that the image data can be transmitted to the consultation experts without distortion. Inefficient web-based reading will lead to long waiting periods, resulting in uncontrollable average visit time. The smooth reading of required data is not only a powerful guarantee for the rapid completion of services, but also the basis for the continuous demand for collaborative medical services.

On the basis of the studies by DeLone and McLean [47] and Taylor and Todd [48], the transaction efficiency of medical services was measured with 6 items that reflect the extent to which the platform provides reliability, timeliness, and comprehensiveness of expert information, as well as timeliness and stability of communication.

#### Big Data Resources

To ensure content validity, the measures for most constructs were used, expanded, and modified from the studies by DeLone and McLean [47], Taylor and Todd [48], Bailey and Pearson [49], and Goodhue [50]. For constructs unique to the big data resources for MCNs, items were self-developed.

The real-time data of diagnosis and treatment services were measured by 3 items that reflect the extent of timeliness, accuracy, and accessibility of physician suspending the diagnosis and treatment as well as the opening information provided by medical institutions to the external systems. The medical service capacity available for external use was measured with 4 items that reflect the extent of the ability and accuracy of external consultation and appointment services provided by medical institutions according to the physician’s workload. The encryption security of web-based data was measured by 4 items that reflect the extent of effect, convenience, transmission efficiency, and coverage of the encryption and decryption technology used by medical institutions when interacting with external systems. The network protection of external link systems was measured with 4 items that reflect the extent of effect, convenience, satisfaction, and coverage of network protection and application protection deployed by medical institutions.

The sharing of diagnosis and treatment data with other medical institutions was measured by 7 items that reflect the extent of accessibility, accuracy, and integrity of diagnosis and treatment data of other medical institutions, as well as the effect of the data sharing on effectively shortening diagnosis time, avoiding repeated examination, avoiding repeated medication, and avoiding adverse drug-drug reactions. Sharing of medical research data with other medical institutions was measured by 3 items that reflect the extent of convenience, functional completeness, and accuracy of research data provided by other medical institutions. The policies and regulations were measured by 3 items that reflect the extent of rationality, existence, and functional completeness of relevant policies, laws, and regulations supporting the construction of a regional medical service platform.

### Data Collection and Demographic Profiles

Data were collected using a survey questionnaire. In China, public hospitals are the main institutions providing health care services. Accordingly, we mainly chose public hospitals, along with some private hospitals. It is very important for medical staff to cooperate closely with IT staff to ensure the implementation of collaborative medical services. Accordingly, each medical institution selected 1 medical staff member and 1 IT staff member as respondents.

The specific data collection plan was designed as follows:

1. Contact the relevant personnel at the target medical institution through WeChat and ask whether they were willing to participate in the survey.
2. Through the relevant personnel, ask the medical institution to determine the respondents, administer the questionnaire on-site, and collect it after completion.
3. If the medical institution is located far away and if the relevant person agrees, provide the questionnaire through WeChat to the person responsible for administering it.

The survey packages were mailed to the appropriate IT executive at each target hospital, with a request that the recipient complete the survey.

The survey packages were also mailed to the appropriate business executive at each target hospital. Part A of the questionnaire was distributed among the appropriate medical staff to complete the measurement items regarding sharing of diagnosis and treatment data, sharing of research data, and transaction efficiency of medical services. Part B was distributed among the appropriate IT executive staff to complete the items related to real-time data of diagnosis and treatment services, medical service capacity available for external use, network protection of external link systems, encryption security of web-based data, and policies and regulations. The questionnaire was administered between August 1, 2017, and October 31, 2017.

Of the 150 medical institutions (involving 18 provinces, autonomous regions, and municipalities) that participated in the survey, 132 (88%) provided valid questionnaires. A total of 264 respondents took part: 132 (50%) IT staff and 132 (50%) medical staff. The sample profile is shown in Table 1.

**Table 1:** Statistical description of the sample (N=132).

Variables and categories				Values, n (%)
**Hospitals**				
	**Hospital level**			
		Tertiary general hospitals	39 (29.5)	
		Tertiary specialty hospitals	15 (11.4)	
		Second-class general hospitals	75 (56.8)	
		Second-class specialty hospitals	4 (3)	
		Community hospitals	1 (0.8)	
	**Type of hospital**			
		Public hospitals	126 (95.5)	
		General practice	6 (4.5)	
**IT^a^ staff**				
	**Sex**			
		Male	83 (62.9)	
		Female	49 (37.1)	
	**Age (years)**			
		20-30	35 (26.5)	
		31-40	79 (59.8)	
		41-50	18 (13.6)	
	**Education**			
		High school graduate	8 (6.1)	
		Bachelor’s degree	114 (86.4)	
		Master’s degree	10 (7.6)	
**Medical staff**				
	**Sex**			
		Male	74 (56.1)	
		Female	58 (43.9)	
	**Age (years)**			
		20-30	23 (17.4)	
		31-40	72 (54.5)	
		41-50	30 (22.7)	
		51-60	7 (5.3)	
	**Education**			
		High school graduate	5 (3.8)	
		Bachelor’s degree	75 (56.8)	
		Master’s degree	50 (37.9)	
		Doctorate	2 (1.5)	

^a^IT: information technology.

### Ethics Approval

The study protocol was reviewed and approved by the ethics review committee at the Shanghai Chest Hospital (IS[P]22003). Before the research was conducted, all participants gave their consent in writing after being informed of the purpose and procedure of the study. We ensured the confidentiality and anonymity of the information collected from the participants.

### Data Analysis Process

SmartPLS is a component-based path-modeling software tool based on the partial least squares regression method. We used SmartPLS (version 2.0) to evaluate the measurement properties and test our hypotheses. Our strategy for data analysis was as follows. First, we evaluated the measurement model by analyzing reliability and validity (including convergent and discriminant validity). Next, applying SmartPLS by using the standard bootstrap resampling procedure (5000 samples) to estimate the significance of the paths, the direct impact of big data resources on transaction efficiency of medical services was examined. For those big data resources that had no direct impact on transaction efficiency, we analyzed their indirect impact.

## Results

### Reliability and Validity

The measurement model was evaluated using the following criteria:

1. Reliability: The outer loading for the indicator should be ≥0.70 (indicator reliability). The cutoff value for Cronbach α was .70 and that for composite reliability was 0.70 (internal consistency reliability) [51].
2. Validity: The average variance extracted (AVE) should be ≥0.50 (convergent validity), based on the Fornell-Larcker criterion [52] (discriminant validity).

As shown in Table 2, the factor loading values of all items were higher than 0.89 and significant at *P*=.001, with composite reliability value=0.9, above the normal value of 0.7. All values met the minimum requirement for indicator reliability and internal consistency reliability. In addition, the AVE used to assess the convergent validity was >0.70 for all constructs, proving that the model had good convergence validity.

**Table 2:** Reliability and convergence validity test results.

Constructs and items		Values, mean (SD)	Load value	Composite reliability		Average variance extracted	
**Encryption security of** **web-based** **data**					0.970		0.891
	ES^a^_1	4.99 (1.532)	0.959				
	ES_2	4.95 (1.536)	0.928				
	ES_3	5.09 (1.395)	0.951				
	ES_4	4.85 (1.515)	0.937				
**Network protection of external link systems**					0.961		0.862
	NP^b^_1	5.72 (1.236)	0.912				
	NP_2	5.69 (1.253)	0.941				
	NP_3	5.51 (1.224)	0.944				
	NP_4	5.47 (1.383)	0.916				
**Real-time data of diagnosis and treatment services**					0.995		0.983
	RT^c^_1	5.15 (1.619)	0.990				
	RT_2	5.11 (1.644)	0.995				
	RT_3	5.11 (1.611)	0.991				
**Medical service capacity available for external use**					0.995		0.982
	SC^d^_1	4.33 (1.812)	0.991				
	SC_2	4.30 (1.788)	0.992				
	SC_3	4.44 (1.798)	0.988				
	SC_4	4.31 (1.781)	0.993				
**Policies and regulations related to big data**					0.956		0.879
	PR^e^_1	5.5 (1.297)	0.968				
	PR_2	5.64 (1.151)	0.919				
	PR_3	5.33 (1.292)	0.925				
**Sharing of diagnosis and treatment data**					0.990		0.931
	TS^f^_1	4.4 (1.654)	0.964				
	TS_2	4.57 (1.687)	0.950				
	TS_3	4.39 (1.681)	0.973				
	TS_4	4.54 (1.656)	0.958				
	TS_5	4.47 (1.820)	0.972				
	TS_6	4.56 (1.715)	0.968				
	TS_7	4.45 (1.836)	0.970				
**Sharing of medical research data**					0.984		0.952
	RS^g^_1	4.66 (1.690)	0.966				
	RS_2	4.82 (1.587)	0.978				
	RS_3	4.79 (1.717)	0.984				
**Transaction efficiency of medical services**					0.973		0.859
	TE^h^_1	4.84 (1.621)	0.937				
	TE_2	4.92 (1.574)	0.947				
	TE_3	4.89 (1.580)	0.953				
	TE_4	4.91 (1.551)	0.925				
	TE_5	4.86 (1.528)	0.906				
	TE_6	4.95 (1.541)	0.890				

^a^ES: encryption security of web-based data.

^b^NP: network protection of external link systems.

^c^RT: real-time data of diagnosis and treatment services.

^d^SC: medical service capacity available for external use.

^e^PR: policies and regulations.

^f^TS: sharing of diagnosis and treatment data.

^g^RS: sharing of medical research data.

^h^TE: transaction efficiency of medical services.

Table 3 presents the test results of discriminant validity. The square root of the AVE values of each construct were greater than the correlation coefficient between the constructs, which conforms to the Fornell-Larcker criterion [52], proving that the measurement model had good discriminant validity.

**Table 3:** Discriminant validity test results.

	ES^a^	NP^b^	RT^c^	SC^d^	PR^e^	TS^f^	RS^g^	TE^h^
ES	0.944	—^i^	—	—	—	—	—	—
NP	0.540	0.928	—	—	—	—	—	—
RT	0.475	0.613	0.992	—	—	—	—	—
SC	0.690	0.432	0.615	0.991	—	—	—	—
PR	0.637	0.601	0.527	0.658	0.938	—	—	—
TS	0.359	0.286	0.423	0.417	0.346	0.965	—	—
RS	0.430	0.318	0.508	0.433	0.521	0.698	0.976	—
TE	0.466	0.527	0.554	0.500	0.519	0.581	0.621	0.927

^a^ES: encryption security of web-based data.

^b^NP: network protection of external link systems.

^c^RT: real-time data of diagnosis and treatment services.

^d^SC: medical service capacity available for external use.

^e^PR: policies and regulations.

^f^TS: sharing of diagnosis and treatment data.

^g^RS: sharing of medical research data.

^h^TE: transaction efficiency of medical services.

^i^Not applicable.

### Influence Path

#### Overview

**Figure 2:**
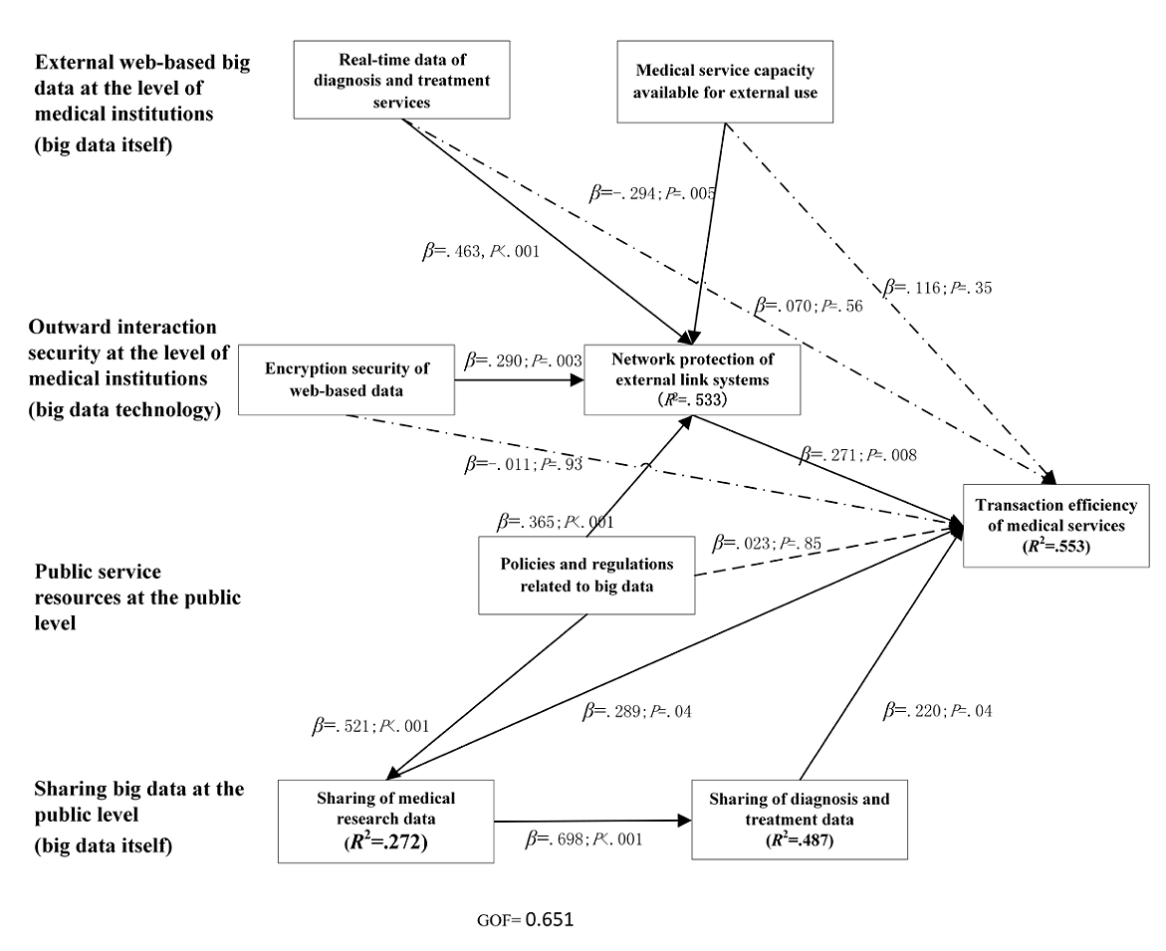
Model results, including direct and indirect effects. GOF: goodness of fit.

The results of the influence path analysis, including the standardized regression weights and levels of significance, are presented in Table 4 and Figure 2. The coefficient of determination *R*^2^ was used to measure the explained variance of the latent dependent variables compared with the total variance. The cutoff levels were as follows: 0.190, weak; 0.333, moderate; and 0.670, substantial; 55.3% of the variance in transaction efficiency of medical services, 53.3% of the variance in the network protection of external link systems, and 48.7% of the variance in sharing of diagnosis and treatment data were moderately explained, whereas 27.2% of the variance in sharing of medical research data was weakly explained, but met the cutoff level.

**Table 4:** Direct effect test results.

Hypothesis	Direct path	*β* coefficient (SE)	*P* value	Support
H1a	RT^a^ to TE^b^	.070 (0.121)	.56	Not supported
H1b	SC^c^ to TE	.116 (0.123)	.35	Not supported
H2a	ES^d^ to TE	−.011 (0.115)	.93	Not supported
H2b	NP^e^ to TE	.271 (0.101)	.008	Supported
H3a	TS^f^ to TE	.220 (0.105)	.03	Supported
H3b	RS^g^ to TE	.289 (0.135)	.04	Supported
H4	PR^h^ to TE	.023 (0.118)	.85	Not supported

^a^RT: real-time data of diagnosis and treatment services.

^b^TE: transaction efficiency of medical services.

^c^SC: medical service capacity available for external use.

^d^ES: encryption security of web-based data.

^e^NP: network protection of external link systems.

^f^TS: sharing of diagnosis and treatment data.

^g^RS: sharing of medical research data.

^h^PR: policies and regulations.

The model’s goodness of fit was our last criterion to assess the overall fit of the model. The model’s goodness of fit for this study as calculated was 0.651, which was deemed large [53].

#### Direct Influence Path

From Figure 2, it can be observed that the direct effects of the network protection of external link systems (*β*=.271; *P*=.008), sharing of diagnosis and treatment data (*β*=.220; *P*=.04), and sharing of medical research data (*β*=.289; *P*=.04) on transaction efficiency of medical services were significant. Hypotheses H2b, H3a, and H3b gained empirical support.

The direct effects of real-time data of diagnosis and treatment services, medical service capacity available for external use, encryption security of web-based data, and policies and regulations on transaction efficiency of medical services were not significant. Hypotheses H1a, H1b, H2a, and H4 did not gain empirical support.

#### Indirect Influence Analysis

As the encryption security of web-based data, real-time data of diagnosis and treatment services, medical service capacity available for external use, and policies and regulations had no direct impact on transaction efficiency of medical services, the indirect effects of these 4 variables on transaction efficiency of medical services were further analyzed. The results of the mediation test are presented in Table 5 and Figure 2. To assess the magnitude of the indirect effect [54], the variance accounted for (VAF) value was calculated, which represents the relationship between the indirect effect and the total effect.

From Table 5, we can observe the following:

1. The indirect impact of policies and regulations. Although the policies and regulations had no direct impact on transaction efficiency of medical services, there was a completely mediated path (policies and regulations → network protection of external link systems → transaction efficiency of medical services) in which the network protection of external link systems played a mediating role in the effect of policies and regulations on transaction efficiency of medical services (VAF=0.945; *P*=.03). It indicated that the government’s establishment of regulations in network security should be conducive to ensuring transaction efficiency and data security.
2. The indirect impact of the encryption security of web-based data. Although the encryption security of web-based data in the external web-based security environment had no direct impact on the transaction efficiency of medical services, there was a completely mediated path (encryption security of web-based data → network protection of external link systems → transaction efficiency of medical services) in which the network protection of external lnk systems played a mediating role in the effect of encryption security of web-based data on transaction efficiency of medical services (VAF=0.879; *P*=.03). It indicated that the encryption security of web-based data improved people’s perception of the degree of network protection of external systems and indirectly affected the transaction efficiency of medical services.
3. The indirect impact of real-time data of diagnosis and treatment services. Although the real-time data of diagnosis and treatment services in the external big data analysis environment had no direct impact on the transaction efficiency of medical services, there was a completely mediated path (real-time data of diagnosis and treatment services → network protection of external link systems → transaction efficiency of medical services) in which the network protection of external link systems played a mediating role in the effect of real-time data of diagnosis and treatment services on transaction efficiency of medical services (VAF=0.678; *P*=.02). It indicated that the stronger the ability of internal data extraction, the safer the external data pipeline and the higher the transaction efficiency.
4. The indirect impact of medical service capacity available for external use. Although the medical service capacity available for external use in the external big data analysis environment had no direct impact on the transaction efficiency of medical services, there was a completely mediated path (medical service capacity available for external use → network protection of external link systems → transaction efficiency of medical services) in which there was the indirect effect of medical service capacity available for external use through the network protection of external link systems on transaction efficiency of medical services (VAF=0.391; *P*=.05). From Figure 2, it can be observed that medical service capacity available for external use has a significant direct negative effect on the network protection of external link systems (*β*=−0.294; *P*=.005), which indicated that frequent service adjustment will increase the complexity of security control and indirectly reduce the transaction efficiency of medical services.
5. The indirect impact of sharing of medical research data. In addition to the direct and significant impact of medical services on the transaction efficiency, there was a partial mediated path (sharing of medical research data → sharing of diagnosis and treatment data → transaction efficiency of medical services) in which sharing of diagnosis and treatment data played a mediating role in the effect of sharing of medical research data on transaction efficiency of medical services (VAF=0.345; *P*=.04). The sharing of research data was conducive to the ability of physicians to interpret the patient’s past medical history to issue an accurate diagnosis faster, promote the sharing of diagnosis and treatment data, and indirectly promote transaction efficiency of medical services.

**Table 5:** Mediation test results.

Indirect effect/direct path		*P* value	Mediated paths		Sobel test					VAF^a^		Type of relationship
					Sobel statistic (SE)		*P* value					
**PR^b^ to TE^c^**				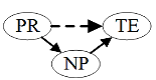		2.170 (0.046)		.03		0.945		Full mediation
	PR to TE	.85									
	PR to NP^d^	<.001									
	NP to TE	.008									
**ES^e^ to TE**				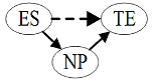		2.122 (0.042)		.03		0.879		Full mediation
	ES to TE	.93									
	ES to NP	.003									
	NP to TE	.008									
**RT^f^ to TE**				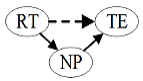		2.313 (0.054)		.02		0.678		Full mediation
	RT to TE	.56									
	RT to NP	<.001									
	NP to TE	.008									
**SC^g^ to TE**				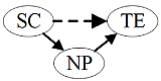		−1.958 (0.041)		.05		0.391		Full mediation
	SC to TE	.35									
	SC to NP	.005									
	NP to TE	.008									
**RS^h^ to TE**				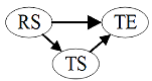		2.086 (0.075)		.04		0.345		Partial mediation
	RS to TE	.04									
	RS to TS^i^	<.001									
	TS to TE	.04									

^a^VAF: variance accounted for.

^b^PR: policies and regulations.

^c^TE: transaction efficiency of medical services.

^d^NP: network protection of external link systems.

^e^ES: encryption security of web-based data.

^f^RT: real-time data of diagnosis and treatment services.

^g^SC: medical service capacity available for external use.

^h^RS: sharing of medical research data.

^i^TS: sharing of diagnosis and treatment data.

## Discussion

### Principal Findings

On the basis of the assumption that MCNs are endogenous and that service transaction efficiency is the key variable for the emergence and development of MCNs, this study empirically analyzed the impact of big data resources of MCNs on the transaction efficiency of health care and provided evidence regarding the following:

1. Sharing of diagnosis and treatment data (big data itself) at the network level directly affected the transaction efficiency of medical services.
  An important challenge of implementing precision medicine based on big data is to share data in MCNs [45]. Sharing diagnosis and treatment data with other hospitals or institutions is an important part of the big data–sharing environment [41]. Only by formulating the classification, grading, and domain-sharing system of medical big data can we steadily promote the opening of medical big data. The sharing of diagnosis and treatment data can result in many obvious benefits, including timely and effective improvement in diagnosis accuracy, strengthening of physician-patient communication and coordination, reduction in repeated treatments, and decrease in the risk of medical errors. By accessing the entire treatment record of the patient through government or third-party platforms, physicians can quickly review the patient’s condition, reduce medical expenses, and avoid adverse medical events such as drug-drug interactions and drug contraindications, thus improving the overall transaction efficiency of medical services.
2. Sharing of research data (big data itself) at the network level directly affected the transaction efficiency of medical services.
  The sharing of research data is another important factor in the overall improvement of medical service quality. Be it clinical effectiveness research, new drug development, or basic medical research, each is often based on the research results of others [40,55]. There are already many shared and free medical research databases such as the electrocardiogram database of the National Institutes of Health, Brain-CODE [43], and Alzheimer disease big data [56] that have advanced related medical research. Integrating the research data of multiple medical institutions is conducive to overcoming the limitations of scientific research and improving the scientific research ability of physicians. With the advent of the era of precision medicine, more and more knowledge-sharing methods have come into being, which has promoted the improvement of multidisciplinary diagnosis and treatment ability and improved the transaction efficiency of medical services.
3. Network protection of external link systems (big data technologies) at the level of medical institutions directly affected the transaction efficiency of medical services.
  Outward interaction security (big data technologies) at the level of medical institutions provides a safe and efficient web-based environment in which a medical institution can be connected with other hospitals or institutions and exchange data. To connect data distributed in different medical institutions steadily and continuously, the first thing to address is the security problem [7,31,57].
  In the past, medical institutions only needed to pay attention to the security of the internal network, which was basically isolated from the outside world. The local area network had high security but poor interoperability. With the development of the internet and big data, the applications of telemedicine are changing rapidly [23,24] and medical institutions are facing increasing need for connections to other hospitals or institutions. The network protection of an outreach system is an important security guarantee for contact between medical institutions and the outside world. Network protection must take into account both security and efficiency, and it should not reduce the efficiency and availability of facilities while ensuring the security of data exchanged by external systems. Abbasi et al [58] point out that through a secure and stable link, the activities of the cooperating parties in the network can be more closely linked and the transaction is more efficient.
4. Real-time data of diagnosis and treatment services (big data itself), medical service capacity available for external use (big data itself), encryption security of web-based data (big data technologies) at the level of medical institutions, and policies and regulations at the network level indirectly affected the transaction efficiency of medical services through network protection of the outreach system (big data technology) at the level of medical institutions. These 4 big data resources will affect the perception of physicians regarding the deployment of a physical security foundation for the connection between medical institutions and the outside world [24,44]. These results highlight that big data technology, big data, and policy at the network and organizational levels interact with, and influence, each other to form the service transaction efficiency of various MCNs.

### Theoretical Implications

This study contributes to research in 3 ways. First, we highlighted the important role of service transaction efficiency in MCN research. Prior research has largely emphasized that service transaction efficiency is one of the factors that affect the operation effect of specific MCNs [6]. In these studies, it was often assumed that MCNs are exogenous and that there is an absolute standard for the quality of MCNs. But this paper emphasized that an MCN is not exogenous; rather, many factors are responsible for its emergence and development. On the basis of the theory of neoclassical economics [27], this study took service transaction efficiency as the key variable for the emergence and development of MCNs and connected the 2 perspectives of factor-oriented research and process-oriented research in current collaborative medical research. From the perspective of MCN being endogenous, the foothold of the study was not the absolute quality of the MCN but the fitness of the MCN to the specific environment. On the basis of transaction efficiency, the study provided the basis for future research on the emergence and development of MCNs. This logic may help explain why there are various contradictions in prior studies on the factors responsible.

Second, we conceptualized big data resources oriented to MCNs from the network and medical institution levels, including big data itself, big data technology, and policy. The combination of big data resources at the level of medical institutions in the MCN and the network public level of the MCN thus affected the transaction efficiency of medical services as a key variable for the emergence and development of MCNs. It emphasized the coexistence and intertwined influence of public big data resources of MCNs and private big data resources in MCNs. This study expanded the limitation of the existing IT-enabling value based on the resource-based view of the firm, which emphasized the private and exclusive nature of IT resources. It also corresponded to the call for research on analyzing the value realization of big data from the work practice, organizational, and supraorganizational levels [17].

Third, this study provided empirical support for De Mauro et al [18] and Wamba et al [19], who proposed integrating big data technology, big data itself, and policy to realize the value of big data. The results further refined and enriched this insight to reveal the detailed impact path of big data technologies, big data itself, and policies on transaction efficiency of medical services. Big data itself was divided into the network level and the organizational level. Big data assets at the network level have a direct impact on transaction efficiency of medical services. However, big data assets at the organizational level affected the transaction efficiency by affecting people’s perception of outward interaction security technology at the organizational level. The negative impact of medical service capacity available for external use on network protection of external link systems indicated that an increase in external services would make people develop a great sense of insecurity. Policies and regulations related to big data at the public level cannot directly affect the services’ transaction efficiency, but they affected the overall formation and operation of MCNs by affecting the public big data resources and the perception of outward interaction security technology at the organizational level.

### Practical Implications

The results have several implications for practice. This study provided the corresponding theoretical guidance for the government to formulate policies. The government should specify corresponding strategies to develop policies regarding sharing of big data resources at the public level and promote various institutions to strengthen the security of external collaborative networks. These policies will affect the ecological service environment of an MCN’s operation to improve transaction efficiency and ultimately enhance the development of MCNs. In addition, all kinds of medical institutions that are willing to interact with the outside world to form an MCN must first strengthen network security, which can especially balance the negative effects caused by the increase in external collaborative services.

### Study Limitations

This study includes several limitations. The data collection was based on the convenient sampling method. Although the medical institutions covered were basically in line with the relative proportion of public and private hospitals in China’s medical institutions, the selection of regions was based on the principle of convenient sampling. Furthermore, this study only considered the transaction efficiency of medical services to reveal the impact of big data resources on the emergence and development of MCNs. In fact, other variables, such as the learning cost of medical services, can affect the emergence and development of MCNs. Future research can analyze the impact of big data resources on the emergence and development of MCNs from the perspective of the learning cost of medical services.

### Conclusions

Our study contributes to both theory and practice. First, it focused on the effects of big data resources on the transaction efficiency of medical services and highlighted how MCNs emerge and develop. Second, it theorized that there are two levels of big data resources—network level and medical institution level—and highlighted the intertwined effect of public and private big data resources on transaction efficiency (including direct impact and intermediary impact). Third, it focused on the effects of health care big data itself, big data technology, and policy on transaction efficiency and revealed the interaction and influence mechanism of these 3 elements of big data value as well as their impact on the formation and development of MCNs.

## Appendix

Multimedia Appendix 1 Questionnaire items.

## Abbreviations

AVE: average variance extracted
IT: information technology
MCN: medical collaborative network
VAF: variance accounted for
