# Supplementary material for: Mechanism of Impact of Big Data Resources on Medical Collaborative Networks From the Perspective of Transaction Efficiency of Medical Services: Survey Study
Source: J Med Internet Res. 2022 Apr 21;24(4):e32776. doi: 10.2196/32776 (PMC9073602; doi:10.2196/32776)
Supplement: Multimedia Appendix 1 [file jmir_v24i4e32776_app1.docx]

# Appendix 1: Questionnaire items

**Part A: Questionnaire items for medical staff**

***Sharing of diagnosis and treatment data (TS)***

TS_1: Diagnosis and treatment data of other medical institutions can be easily obtained (such as data sharing through government public platforms and medical networks).

TS_2: The information provided by other medical institutions' diagnosis and treatment data sharing (such as through government public platforms, medical networking) is accurate.

TS_3: Diagnosis and treatment data sharing of other medical institutions (such as through government public platforms, medical networking) provides more comprehensive information.

TS_4: The diagnosis and treatment data sharing of other medical institutions (such as through government public platforms, medical networking) effectively reduces the time of the diagnosis time of diseases.

TS_5: The diagnosis and treatment data sharing of other medical institutions (such as through government public platforms, medical networking) avoids repeated inspections.

TS_6: The diagnosis and treatment data sharing of other medical institutions (such as through government public platforms, medical networking) avoids repeated medication.

TS_7: Diagnosis and treatment data sharing of other medical institutions (such as through government public platforms, medical networking) avoids adverse reactions between various drugs.

***Sharing of medical research data (RS)***

RS_1: Research data from other medical institutions (CNKI, PubMed, etc.) can be easily obtained.

RS_2: The research data sharing of other medical institutions (CNKI, PubMed, etc.) provides relatively complete scientific research information.

RS_3: Research data from other medical institutions (CNKI, PubMed, etc.) are all valuable.

***Transaction efficiency of medical services (TE)***

TE_1: On these third-party platforms (such as BaiYulan, Cloud Hospital, etc.), the consultation expert information provided by our hospital is accurate.

TE_2: On these third-party platforms, the consultation expert information provided by our hospital (including the introduction and professionality of the expert) is updated in real time.

TE_3: On these third-party platforms, the information of consultation experts provided by our hospital is very comprehensive.

TE_4: The consultation platform of our hospital provides a better display page, in which the patients’ objective medical history can be check at a glance.

TE_5: The consultation platform of our hospital is very fast when accessing patients' medical history and imaging data or video conversations.

TE_6: The consultation platform of our hospital is very stable when consulting patient information or video conversations.

**Part B: Questionnaire items for IT staff**

***Real-time data of diagnosis and treatment services (RT)***

RT_1: Our hospital’s information system can provide timely information on doctors’ suspension and opening consultations for external systems (such as remote consultation platforms, government public platforms, and medical networking).

RT_2: The information system of our hospital can accurately provide information on doctors' suspension and opening of consultations for external systems (such as remote consultation platforms, government public platforms, and medical networking).

RT_3: The information system of our hospital can conveniently provide information such as doctors' suspension and opening consultations for external systems (such as remote consultation platforms, government public platforms, and medical networking).

***Medical service capacity available for external use (SC)***

SC_1: The information system of our hospital can determine the doctor's external consultation service according to the doctor's workload.

SC_2: The information system of our hospital can determine the doctor's external appointment service according to the doctor's workload.

SC_3: The information system of our hospital can accurately determine the doctor's external appointment service according to the doctor's workload.

SC_4: The information system of our hospital can accurately determine the doctor's external consultation services according to the doctor's workload.

***Encryption security of web-based data (ES)***

ES_1: Our hospital uses encryption and decryption technology to exchange data with external systems, and the effect is good.

ES_2: In the interaction with the external system of the hospital, the data transmission encryption and decryption technology did not increase my workload.

ES_3: The process of adopting data transmission encryption and decryption technology in our hospital has not affected the efficiency of data transmission with the external systems of the hospital.

ES_4: Our hospital has used data transmission encryption and decryption technology in all interactions with the external system of the hospital.

***Network protection of external link systems (NP)***

NP_1: Our hospital did not maliciously damage external systems (such as remote consultation platform, government public platform, medical network) when using network security (website protection, APP protection) tools.

NP_2: When our hospital uses network security (website protection, APP protection) tools, it does not cause any inconvenience to our use of external systems (such as remote consultation platforms, government public platforms, and medical networking).

NP_3: When our hospital uses various external systems (such as remote consultation platform, government public platform, medical network) with network security protection (website protection, APP protection), it feels very smooth.

NP_4: In my daily work, various external systems (such as remote consultation platforms, government public platforms, and medical networking) that I come into contact with have a network security foundation (website protection, APP protection).

***Policies and regulations (PR)***

PR_1: The relevant policies, laws and regulations on the regional medical service platform (three-level referral from the Health and Family Planning Commission, medical consortium, and regional medical treatment) are reasonable.

PR_2: The construction of the regional medical service platform is supported by policies, laws and regulations (three-level referral from the Health and Family Planning Commission, medical consortium, and regional medical treatment).

PR_3: The difficulties encountered in the construction of the regional medical service platform can be solved according to policies, laws and regulations (three-level referral from the Health and Family Planning Commission, medical consortium, and regional medical treatment).
